# Clinical psychomotor skills among left and right handed medical students: are the left-handed medical students left out?

**DOI:** 10.1186/s12909-016-0611-7

**Published:** 2016-03-22

**Authors:** Sami Alnassar, Aljoharah Nasser Alrashoudi, Mody Alaqeel, Hala Alotaibi, Alanoud Alkahel, Waseem Hajjar, Ghadeer Al-shaikh, Abdulaziz Alsaif, Shafiul Haque, Sultan Ayoub Meo

**Affiliations:** Department of Thoracic Surgery and Department of Medical Education, Riyadh, Saudi Arabia; Department of Medical Education, Riyadh, Saudi Arabia; Department of Obstetrics and Gynecology, Riyadh, Saudi Arabia; Department of Surgery, Riyadh, Saudi Arabia; Department of Medical Education, Riyadh, Saudi Arabia; Department of Physiology, College of Medicine, King Saud University, Riyadh, Kingdom of Saudi Arabia; Thoracic Surgery, Department of Surgery (37) and Department of Medical Education, College of Medicine, King Saud University, PO Box 7805, Riyadh, 11472 Saudi Arabia

**Keywords:** Clinical psychomotor skills left-handed, Right handed

## Abstract

**Background:**

There is a growing perception that the left handed (LH) medical students are facing difficulties while performing the clinical tasks that involve psychomotor skill, although the evidence is very limited and diverse. The present study aimed to evaluate the clinical psychomotor skills among Right-handed (RH) and left-handed (LH) medical students.

**Methods:**

For this study, 54 (27 left handed and 27 right handed) first year medical students were selected. They were trained for different clinical psychomotor skills including suturing, laparoscopy, intravenous cannulation and urinary catheterization under the supervision of certified instructors. All students were evaluated for psychomotor skills by different instructors. The comparative performance of the students was measured by using a global rating scale, each selected criteria was allotted 5-points score with the total score of 25.

**Results:**

There were no significant differences in the performance of psychomotor skills among LH and RH medical students. The global rating score obtained by medical students in suturing techniques was: LH 15.89 ± 2.88, RH 16.15 ± 2.75 (*p =* 0.737), cannulation techniques LH 20.44 ± 2.81, RH 20.70 ± 2.56 (*p =* 0.725), urinary catheterization LH 4.33 ± 0.96 RH 4.11 ± 1.05 (*p =* 0.421). For laparoscopic skills total peg transfer time was shorter among LH medical students compared to RH medical students (LH 129.85 ± 80.87 s vs RH 135.52 ± 104.81 s) (*p =* 0.825). However, both RH and LH students completed their procedure within the stipulated time.

**Conclusions:**

Among LH and RH medical students no significant difference was observed in performing the common surgical psychomotor skills. Surgical skills for LH or RH might not be a result of innate dexterity but rather the academic environment in which they are trained and assessed. Early laterality-related mentoring in medical schools as well as during the clinical residency might reduce the inconveniences faced by the left-handed medical personnel.

## Background

The current world population is about 7.2 billion and 10 % of the global population is left handed (LH) [1]. Some people believe that being LH is a blessing or even it is considered as a sign of intellectual [2] or creativity [3–5]. It is also considered a major disability and a social disgrace. Historical evidence shows that left-handedness is sometimes regarded as social stigma. The majority of the world population is right handed (RH) and LH persons are in a minority, especially in the surgical field and this is endorsed by the fact that none of the medical science textbook and tools narrates for LH medical students. An earlier report shows that, out of ten medical personnel, one is LH [6]. Due to RH majority, most of the surgical instruments and apparatuses are designed for RH medical personnel, the locking and unlocking actions for the needle holders have also been designed for RH surgeons [7–9].

LH surgeons have several disadvantages in the field of surgery, where they always need supporting personnel to assist them and they are required to adapt themselves to the given environment [10]. The LH surgeons must go through the surgical procedures as defined by the RH surgeons and have to add needful modifications to practice the safe and convenient procedures [11]. There is a general impression among medical community that LH practitioners face difficulties while performing some basic procedures or during some delicate surgical operations wherein all apparatus, medical equipment and materials have been designed for the right handed (RH) population. Some reports are available related to the impact of hand dominance on psychomotor skills [7, 12–15]. Most of them are based on surveys conducted among mentors or surgeons and post-training assessment [6, 16–26], although, the evidence is very limited and diverse. Considering aforesaid facts, the present study aimed to evaluate and compare the clinical psychomotor skills among RH and LH medical students.

## Methods

### Study design and settings

The present cross sectional study was conducted in the Department of Medical Education, Clinical Skills and Simulation Center, College of Medicine, King Saud University, Riyadh, Saudi Arabia during the period 2012 to 2014.

### Students’ selection

In this study, we selected 54 first year undergraduate medical students. All the students were selected based on their voluntary participation, same age (20.23 ± 0.68 years); gender, academic class, nationality, regional and cultural background and with similar pervious performance in their academic grades. Out of 54 first year undergraduate medical students, 27 students were LH (14 females and 13 males) and 27 were RH students (14 females and 13 males). The selected students were trained for different clinical psychomotor skills under the supervision of instructors. The instructors were board certified fellows working in level-1 accredited clinical skills and simulation center. The selected trained students were evaluated for the same skills by other instructors (examiners). All students signed the informed written consent.

### Exclusion criteria

All the students were checked for previous knowledge, trainings on clinical psychomotor skills and any physical injury of the hand. Students with different level of mental and scientific knowledge were excluded from the study in order to avoid the selection bias and corresponding dubious results. Students who suffered from any chronic illness, anemia and diabetes mellitus were excluded from the study, as these diseases are known to affect cognitive functions such as attention, understanding, and producing [27]. Moreover, students who were either outstanding or failing in their previous examination were also excluded to minimize differences of knowledge and skills [28].

### Faculty involvement

Senior faculty members and consultants were involved in the training sessions for the students. Faculty members were briefed about the study protocol and a peer from the Department of Medical Education also ensured that the faculty members were well aware of the system.

### Basic surgical skill course

All selected students (LH and RHs) attended basic surgical skills course and they were not informed about evaluation on the basis of their laterality. To avoid the study bias, the students, instructors and examiners were not informed about the research hypothesis. All participants (LH and RH) were pooled together and assigned into sub groups and it was ensured that each group must include LH and RH students. It was also ensured that none of the participants were aware about their laterality as well as other characteristics during this study except the investigators. Each participating student was assigned a unique code consisting of a letter followed by two numerical digits. The letter corresponded to the selected group, whereas, the numbers corresponded to the ‘participant’. The given codes were noted by the investigators prior to the start of the skills training and remained confidential throughout the study. Each group underwent two days training. Each training station had an assigned instructor to teach and guide the students.

### Day-1 training

One week before the training, all the students were informed about their training program with a detailed schedule. On the first day of training, the students were trained on suturing techniques for 3 h, Intravenous (IV) cannula insertion for 1.5 h, and urinary catheterization also for 1.5 h. Training was conducted at Clinical Skills and Simulation Center based on the lectures and practical sessions. Students were trained on suturing techniques by certified senior faculty members of the department of surgery. They practiced cannula insertion on mannequins (Nasco Venipuncture training model, Nasco, Modesto, USA), and urinary catheterization on mannequins (male and female catheterization simulator, Adam, Rouilly limited, UK). It was also noticed that each participating student used his/her dominant hand to perform the given task whether it was left or right. It was also ensured that all the instruments used for the suturing techniques were of the same type.

### Day-2 training

On the second day of training, each group of students underwent laparoscopy training (laparoscopy simulator, Immersion Corporation, USA) for 3 h. All students learned three skills during the laparoscopy training, i.e., peg transfer skill, clipping skill and cutting skill. The total training duration was 9 h for all the participating students.

### Faculty involvement in objective structured clinical examination (OSCE)

Faculty members and consultants who held postgraduate academic titles were involved in the assessment of the students. For OSPE assessment, two senior faculty members were involved in each group to score the students while assessing the clinical psychomotor skills. In addition, a nonaligned faculty member was involved to ensure the best delivery of the entire assessment. There was a close consensus on the inter-rater reliability among the faculty members. The faculty members who taught the technical skills were different from the evaluators.

### Assessment of skills: objective structured clinical examination (OSCE)

The acquired skills of all the participating students were evaluated through the Objective Structured Clinical Examination (OSCE). All students were assessed by using global rating scale form of skills based evaluation developed by Moorthy et al., [18] and Chipman and Schmitz [29]. OSCE stations with all the essential facilities were set up and students performed the assigned task in the presence of an examiner. Global rating scale was applied, each selected criteria was allotted a 5-points score with the total score of 25 points Moorthy et al., [18]. Students were evaluated on the basis of their surgical knowledge procedure, information and understanding of the instrument, instrument handling, motion and flow of the procedure, and overall performance quality of the technique for the mentioned skills.

### Ethical approval

The ethical and methodological grounds of the study were approved by the Institutional Review Board, College of Medicine, King Saud University, Riyadh, KSA.

### Statistical analysis

Data were entered into the computer and analyzed through Predictive Analytics Software (PASW, version 18.1) program using appropriate statistical tests. The data were tested for normality and continuous variables were analyzed by the student’s *t*-test and categorical variables were evaluated by chi-square test. The level of significance was considered at *p*-value < 0.05.

## Results

This study reveals the clinical psychomotor skills among Right-handed (RH) and left-handed (LH) medical students. The suturing, cannulation techniques, urinary catheterization and laparoscopic skills were assessed.

### (i) Suturing techniques

This study reveals the clinical psychomotor skills among Right-handed (RH) and left-handed (LH) medical students. For procedural knowledge in suturing techniques the rating score of LH medical students was (3.33 ± 0.555) and the RH medical students was (3.44 ± 0.641). The instruments knowledge of LH students was (3.22 ± 0.641) similar to that of RH medical students (3.30 ± 0.609). Moreover, in handling of suturing techniques LH students scored (3.04 ± 0.980) quite close to RH controls (3.15 ± 0.770). Whereas in suturing technique LH medical score was (3.04 ± 0.854) compared to the RH medical students (3.07 ± 0.917). Interestingly, LH students scored higher (3.26 ± 0.712) in the quality of suturing as compared to their RH colleagues (3.19 ± 0.557). Overall, RH students scored higher (16.15 ± 2.755) in total points than LH students (15.89 ± 2.887) (Table 1).

**Table 1:** Performance comparison of LH medical students and RH medical student for suturing techniques (5-points rating score with the total score of 25)

Suturing techniques	LH Subjects (Mean ± SEM) (*n =* 27)	RH controls (Mean ± SEM) (*n =* 27)	Confidence interval (CI)	*p*-value
Procedure knowledge	3.33 ± 0.555	3.44 ± 0.641	−0.438–0.216	0.499
Instrument’s knowledge	3.22 ± 0.641	3.30 ± 0.609	−0.415–0.267	0.665
Handling	3.04 ± 0.980	3.15 ± 0.770	−0.592–0.267	0.645
Motion	3.04 ± 0.854	3.07 ± 0.917	−0.521–0.447	0.879
Overall quality	3.26 ± 0.712	3.19 ± 0.557	−0.275–0.423	0.672
Total score	15.89 ± 2.887	16.15 ± 2.755	−1.8–1.282	0.737

### (ii) Cannulation

In cannulation, LH students scored (4.22 ± 0.641) in knowledge about the procedure while RH students scored (4.04 ± 0.800). Regarding knowledge on IV instruments, both LH (4.04 ± 0.649) and RH students (4.04 ± 0.706) performed similarly. As expected, in instrument handling, LH students scored significantly lower (3.96 ± 0.76) than RH students (4.30 ± 0.465) (*p*-value = 0.04). Hand motions were almost similar for LH (4.07 ± 0.675) and RH (4.07 ± 0.675) students were almost the same. The quality of performance in cannulation and overall scores were higher (4.26 ± 0.594) (20.70 ± 2.569) for RH controls in comparison to LH subjects (4.015 ± 0.662) (20.44 ± 2.819) (Table 2).

**Table 2:** Performance comparison of LH medical students and RH medical students for intravenous cannulation technique (5-points rating score with the total score of 25)

IV cannulation techniques	LH Subjects (Mean ± SEM) (*n =* 27)	RH Controls (Mean ± SEM) (*n =* 27)	Confidence interval (CI)	*p*-value
Procedure knowledge	4.22 ± 0.641	4.04 ± 0.800	−0.213–0.583	0.933
Instrument’s knowledge	4.04 ± 0.649	4.04 ± 0.706	−0.370 ± 0.0370	1.0
Handling	3.96 ± 0.76	4.30 ± 0.465	−0.660–0.07	0.046
Motion	4.07 ± 0.675	4.07 ± 0.675	−0.369 ± 0.369	1.0
Overall quality	4.015 ± 0.662	4.26 ± 0.594	−0.455–0.233	0.519
Total score	20.44 ± 2.819	20.70 ± 2.569	−1.732–1.214	0.725

### (iii) Urinary catheterization

In urinary catheterization skills, LH students’ knowledge regarding procedure scored was (4.04 ± 1.126) while the score of RH students was (4.00 ± 1.00), (*p =* 0.89). Regarding instrument’s knowledge RH medical students scored (3.89 ± 1.050) and LH students scored (4.15 ± 0.808). Similarly, for catheter handling/skills, LH students scored higher (4.11 ± 0.934) than RH controls (3.89 ± 0.974). During urinary catheterization hand motion, LH subjects scored greater (4.26 ± 0.944) than RH controls (4.19 ± 1.001). Overall quality of performance in catheterization was lower (4.11 ± 1.050) for RH students as compared to LH students (4.33 ± 0.961) (Table 3).

**Table 3:** Performance comparison of LH medical students and RH medical students for the urinary catheterization technique (5-points rating score with the total score of 25)

Urinary catheterization	LH Subjects (Mean ± SEM) (*n =* 27)	RH Controls (Mean ± SEM) (*n =* 27)	Confidence interval (CI)	*p*-*value*
Procedure knowledge	4.04 ± 1.126	4.00 ± 1.00	−0.545–0.619	0.899
Instrument’s knowledge	4.15 ± 0.808	3.89 ± 1.05	−0.256–0.773	0.316
Catheter handling	4.11 ± 0.934	3.89 ± 0.974	−0.299–0.743	0.396
Hand motion	4.26 ± 0.944	4.19 ± 1.001	−0.457–0.606	0.417
Overall quality	4.33 ± 0.961	4.11 ± 1.050	−0.327–0.772	0.421
Total score	20.89 ± 1.873	18.28 ± 2.025	−1.627–1.241	0.843

### (iv) Laparoscopic skills

**Fig. 1:**
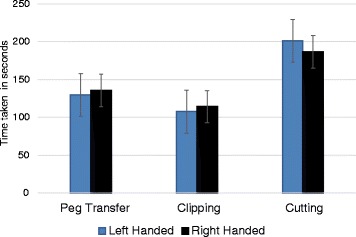
Comparison of laparoscopic skills performance among left handed and right handed medical students during laparoscopy skills training

The total Peg transfer time was shorter among LH medical students as compared to RH medical students [129.85 ± 80.873 s) vs. (135.52 ± 104.815 s), (CI –56.792–45.459) *p*- = 0.825] (Fig. 1). All LH students did not exceed in their task completion time, however, two RH control students failed to complete the task within the stipulated time. All LH student subjects placed their pugs at right place, whereas 20 out of 27 RH students failed. For clipping procedure, LH students took shorter time to complete the procedure while RH students failed to achieve the same [107.58 ± 71.021 s vs. 114.11 ± 71.105 s, (CI –45.403–32.218), *p =* 0.288], (Fig. 1). Both, RH and LH students completed their procedure within the stipulated time. Of the 27 LH students, 16 did not perforate the vessel as compared to 21 out of 27 RH students. On the other hand, 7 out of 27 LH students perforated the duct as compared to 8 out of 27 RH students. In cutting procedure, all students, whether they were LH or RH completed the given task within the stipulated time. Longer cutting time was taken by LH students as compared to RH students [201.33 ± 41.351 s vs. 186.48 ± 42.964 s, (CI –0.344612–1.0818351), *p =* 0.20], (Fig. 1).

## Discussion

The present study illustrates the impact of laterality on clinical psychomotor skills for the undergraduate medical students. The results of this study show that there were no significant differences between LH and RH medical students while performing the clinical psychomotor skills. It was reported in a randomized controlled study that, LH trainees learned bone drilling better with tools designed for left hand whereas left-handers used LH tools and right-handers used RH tools [10]. Adusumilli et al., [6] reported that one out of four LH medical professionals were willing to join a surgical field and the negative impact of left handedness mostly appeared in the early stages of medical career [6]. It is also evident that laparoscopy and laparoscopic instruments failed to eliminate the problems associated with instrument handling for LH surgeons [6, 14, 15].

A survey based study conducted in the Turkish Society of Surgery, showed that about 50 % of LH participating surgeons believed that endoscopic surgery needs to be modified for LH surgeons [25]. Previous study demonstrated lesser errors and better first time accuracy by RH participants for endoscopic manipulation in comparison to LH participants [16]. On the contrary, LH ophthalmic surgical residents had lesser intraoperative complications (e.g., posterior capsule tear and vitreous loss) during cataract surgery than their RH counterparts [12, 17]. Similarly, it has been reported that LH surgical residents are more proficient in a neuropsychological test of tactile-spatial abilities [13]. Case studies of laparoscopic cholecystectomy in situs inversus totalis also reported that RH surgeons experienced more problems while performing the surgery. So, being LH could be an advantage in some surgical events [26]. In case of clinical psychomotor skills, LH surgeons might face some inconvenience while using instruments designed for RH, thus putting safety of the process at risk. Similarly, LH undergraduate medical students may possibly encounter difficulties in managing psychomotor skills training requirements, thus affecting their skills training and career choice.

In this study students’ training was initiated with minor psychomotor skills, like, suturing techniques, cannulation and urinary catheterization which, are usually performed by undergraduate medical students and physicians in all medical fields. On the first day of training, it was noticed that during suturing technique training, some of the students changed their places to sit next to their colleagues who had similar hand preference. Based upon students’ action, it can be hypothesized that it would be bit easier to learn any psychomotor skill when the trainee and the instructor having the similar hand dominance. It was also noticed that some LH students were using their non-dominant right-hand (secondary hand) in performing the skills very nicely, and when we asked them “why don’t you use your dominant hand?” they replied “this is how we learned it when we were taught by the instructors”. It was also noticed during laparoscopy simulator training that some of LH participants used their right hand and most of them were excellent. On the contrary, some of them struggled and asked to switch the tools to fit their hand dominance, hence, failed to perform the skill with right hand.

In the suturing techniques, the mean points for RH trainees were higher than the LH trainees in terms of procedure knowledge, instrument’s knowledge, handling, motion and total score, but the mean score for overall quality was higher for LH trainees though all the differences were statistically insignificant. In the cannulation task, the mean points for RH students were higher than the LH students in terms of handling, overall quality and total score, but LH group scored higher in procedure knowledge and equal points in the motion and instrument’s knowledge. The only statistically significant difference found in terms of the cannulation task was ‘handling’ between the two groups. In the urinary catheterization, the mean points for LH trainees were higher than the mean values for RH trainees in procedure knowledge, motion, overall quality and total score, but the scores were lower for instrument’s knowledge and handling in comparison with their right handed colleagues and all differences were statistically insignificant. It was observed that the ‘knowledge’ was mostly dominated by LH group and they scored higher than the RH group, similarly the instrument handling part was mostly dominated by RH group and they scored higher than LH group.

Lee et al., [30] evaluated the effect of a hand dominance-based curriculum for acquisition of basic suturing and knot tying skills among medical students and demonstrated that there were no significant differences between LH trainees and RH trainees. Both LH and RH trainees were immersed in a training environment that was discordant with their hand dominance. In the present study we also did not find any difference between the LH and RH medical students in performing common surgical psychomotor skills.

During the laparoscopy simulator training, the Metzenbaum scissor was kept on the right hand side and the Straight Grasper on the left hand side. Instruments were not standardized to make it harder for the LH group, because in actual practice of minimally invasive surgery the standard port placement and trocar positioning forces LH professional in some cases to dissect using secondary hand. The outcomes of the present study refuted the well accepted credence that laparoscopy and minimally invasive surgical instruments have totally eradicated problems for the LH medical professionals, which is in agreement with earlier reports of Adusumilli et al., [6]. Generally, laparoscopy occupies the static posture of the neck and the trunk with repeated unusual movements of the upper body part. Thus, in order to facilitate the successful and simple laparoscopy surgery technique, designing and development of dedicated LH instruments might be helpful.

Out of 27 LH medical students only 3 (11.12 %) of them demanded to change the instrument’s place and expressed their left-handedness concerns, it might be possible that the remaining LH students (88.88 %) were also uncomfortable with RH instruments but they did not express probably due to their hesitant nature or reluctance towards learning or negligence due their hectic schedule or rush to finish the practical training as soon as possible etc. Another interpretation can be that only 11.12 % of LH medical students were aware and anxious about their laterality issues and sought advice during training for laterality predominance. Still no provision of laterality preference for the LH students was noted during training, so, prior information and mutual choice about mentor/students laterality preference might eliminate the understanding conflicts and training hassles between mentor and the medical students.

Analysis of the peg transfer skill showed that LH trainees spent shorter time because the peg was placed on left side so it was easier for LH trainees to hold and keep it on right place, thus dexterity is not in favor of RH surgeons [11], like the case of situs inversus [26]. While analyzing the clipping skills data, the numbers of clipping correct clips with left-hand were excluded from the results because the clipper was in right hand, so all participants used their right hand. During analysis of the cutting skills data, the numbers of unsuccessful cutting with the left hand were excluded from the results because the Metzenbaum scissor was in right hand, so all participants used their right hand. LH trainees spent shorter time in clipping and due to that they perforated the vessels and after bleeding the procedure ended. Also, the evaluation results of the laparoscopy simulator training were statistically insignificant [11].

### Study limitations

The major constraint of this study is its smaller sample size, we tried to recruit LH undergraduate medical students of equal mental and technical level who hold good understanding of medical sciences, but practically it is not feasible. Students with different level of mental, technical and scientific knowledge were excluded from the study in order to avoid bias and the corresponding dubious results. Future studies with a larger number of participating medical students with suitable psychometric assessment might help to minimize the above mentioned inadequacies.

## Conclusions

There is no significant difference between LH and RH medical students in performing common surgical psychomotor skills. Surgical skills for LH or RH might not be a result of innate dexterity but rather the academic environment in which they are trained and assessed. Early laterality-related mentoring in medical schools and during the clinical residency might reduce the inconveniences in the left-handed medical personnel learning. This study reveals the perceptions of LH undergraduate medical students in adapting to a RH world. Based on the current findings, we suggest that early laterality related mentoring and awareness in medical colleges and during surgical residency with especial provision of dedicated LH instruments might decrease the complexity and inadequacies of LH students learning and training. It is also suggested that at least basic understanding of laterality should be provided to all the medical undergraduates in the initial stage and proper arrangement should be there in all medical schools for their guidance. Despite being aware about this situation, no pedagogical material is available to teach and train the LH undergraduate medical students. Hence, in order to tackle this laterality issue of medical undergraduates, minor modifications in training strategy as well as adopting laterality driven instruments in undergraduate medical curriculum might work. A dedicated website for LH medical student’s teaching and learning and other LH training videos might create some laterality awareness in students/faculty. Addition of training schedules for LH medical students in their extra-curricular activities or inclusion of laterality related chapter in medical surgical textbooks or specialized manual mentioning the ‘SOP’ (Standard Operating Procedure) specifically for LH students that list the available instruments and working methodology might be beneficial in creating awareness about laterality related difficulties. Identification of problematic skills for LH students and developing specific guidelines for teaching of clinical skills must be based on balanced evidence of their practical utility and local needs and suitability for laterality, and not on technophobic prejudices. So, to reduce the laterality related problems, structured approach of teaching psychomotor skills might be employed to enhance student’s performance by considering their laterality and designing novel pedagogical methodologies. Our study also urges for further prodigal studies in relevance with left-handedness of undergraduate medical students by imparting ambilaterality and ambidexterity training on clinical psychomotor skills of higher technical level and by generating consciousness about laterality and/or use of dedicated LH instruments.
